# Dihydropyridine calcium blockers do not interfere with non-rapid eye movement sleep

**DOI:** 10.3389/fnins.2022.969712

**Published:** 2022-10-19

**Authors:** GoEun Han, Sumire Matsumoto, Javier Diaz, Robert W. Greene, Kaspar E. Vogt

**Affiliations:** ^1^International Institute for Integrative Sleep Medicine (WPI-IIIS), University of Tsukuba, Tsukuba, Japan; ^2^Department of Psychiatry & Neuroscience, Peter O’Donnell Jr. Brain Institute, UT Southwestern Medical Center, Dallas, TX, United States

**Keywords:** non-rapid eye movement wave sleep, delta activity, NREM, voltage gated calcium channel, dihydropyridine, calcium imaging

## Abstract

Non-rapid eye movement (NREM) sleep is tightly homeostatically regulated and essential for survival. In the electroencephalogram (EEG), oscillations in the delta (0.5–4 Hz) range are prominent during NREM sleep. These delta oscillations are, to date, the best indicator for homeostatic sleep regulation; they are increased after prolonged waking and fade during NREM sleep. The precise mechanisms underlying sleep homeostasis and the generation of EEG delta oscillations are still being investigated. Activity-dependent neuronal calcium influx has been hypothesized to play an important role in generating delta oscillations and might be involved in downstream signaling that mediates sleep function. Dihydropyridine blockers of L-type voltage-gated calcium channels (VGCCs) are in wide clinical use to treat hypertension and other cardiovascular disorders and are readily blood-brain-barrier penetrant. We therefore, wanted to investigate their potential effects on EEG delta oscillation and homeostatic NREM sleep regulation in freely behaving mice. *In vivo* two-photon imaging of cortical neurons showed larger spontaneous calcium transients in NREM sleep compared to waking. Application of the dihydropyridine calcium blocker nicardipine significantly reduced cortical calcium transients without affecting the generation of delta oscillations. Nicardipine also did not affect EEG delta oscillations over 24 h following application. The time spent in NREM sleep and NREM episode duration was also not affected. Thus, acute block of calcium entry through L-type VGCCs does not interfere with EEG delta oscillations or their homeostatic regulation, despite prior evidence from calcium channel knockout mice.

## Introduction

In this study, we aimed to test two hypotheses. First, whether an activity-dependent influx of calcium through L-type voltage-gated calcium channels (VGCCs) in cortical neurons is necessary for the generation of the characteristic non-rapid eye movement (NREM) sleep electroencephalogram (EEG) delta oscillations (Tatsuki et al., 2016, 2017; Shi and Ueda, 2018). Second, whether L-type VGCC activity is necessary for the gradual reduction of delta oscillations that occur during normal NREM sleep. Sleep is necessary for survival in all animals with a central nervous system and ensures its proper long-term function (Roth, 2009; Zielinski et al., 2016). Rapid eye movement (REM) and NREM sleep can be distinguished in birds and mammals. NREM sleep is characterized by high activity in the low-frequency EEG delta (0.5–4 Hz) band. These delta oscillations are caused by synchronous transitions of cortical neurons between a hyperpolarized, silent OFF state and a depolarized, active ON state, with each state lasting from a few tens to a few hundred milliseconds (Contreras and Steriade, 1995; Amzica and Steriade, 1998; Destexhe et al., 1999). Recent evidence points to a central role of intracellular calcium in cortical neurons in the generation of ON-OFF transitions (Tatsuki et al., 2016, 2017; Shi and Ueda, 2018); according to this model, ON state action potential firing in cortical neurons triggers calcium influx, the rise in intracellular calcium concentration causes the opening of calcium-dependent potassium channels, inhibiting cortical neurons and triggering ON-OFF transitions; calcium extrusion during OFF state silence closes calcium-dependent potassium channels in cortical neurons and triggers OFF-ON transitions. The power of delta oscillations is, to date, the best marker for the homeostatic regulation of NREM sleep (Aeschbach and Borbely, 1993; Achermann et al., 1993; Borbély and Achermann, 1999; Dijk, 2009). High sleep need (e.g., after prolonged waking) is accompanied by high EEG delta power, and as sleep need is reduced during NREM sleep, delta oscillations fade (Aeschbach et al., 1997; Bjorness et al., 2016; Borbély et al., 2016). The mechanisms responsible for the generation of delta oscillations are still poorly understood. Cortical ON/OFF transitions can be observed in the absence of thalamocortical input (Steriade et al., 1993); however, in physiological NREM sleep, thalamic afferents may trigger and synchronize OFF-ON transitions (Gent et al., 2019). Moreover, it is still unknown whether delta oscillations reduce sleep need and exert the beneficial effects of NREM sleep on the brain or whether they are simply an epiphenomenon of the process.

In an effort to find more measures for successful sleep (i.e., sleep that reduces sleep pressure), several recent studies have investigated the effects of waking and sleep on the neural transcriptome, proteome, and phospho-proteome and have found clear patterns that correlate with sleep need (Wang et al., 2018; Brüning et al., 2019; Noya et al., 2019; Suzuki et al., 2020). However, no direct link has been established between neural activity, which generates delta oscillations, and the signaling that leads to the changes in gene expression, protein, and phospho-protein abundance observed after NREM sleep. An influx of calcium ions into neurons is one of the most established mechanisms linking activity to downstream cellular signals, and it has been hypothesized that this plays a role in slow wave generation and sleep (Llinás and Jahnsen, 1982; Deschênes et al., 1984; Steriade et al., 1985; Domich et al., 1986; Bal et al., 1995; Hughes et al., 2004; Lee et al., 2004; Anderson et al., 2005). A block of L-type VGCCs might therefore interfere with the signaling pathways linking neural activity to sleep need resolution. L-type VGCCs are widely expressed in cortical neurons (Hell et al., 1993) and are responsible for a postsynaptic influx of calcium, which can lead to synaptic plasticity and activity-induced regulation of gene expression (Catterall, 2000; Dolmetsch et al., 2001; Schafe and LeDoux, 2008). Dihydropyridines block L-type VGCCs and cause a significant reduction in activity-dependent calcium influx in neurons (Borst and Sakmann, 1996). They are widely used to treat cardiovascular disorders, mainly hypertension, and typically penetrate the blood-brain barrier (Ritz et al., 2010; He et al., 2018). Sleep disturbances are not frequently reported under dihydropyridine treatment and may be limited to patients suffering from obstructive sleep apnea (Nerbass et al., 2011). However, milder symptoms might have been overlooked. Therefore, we were motivated to investigate the effects of the dihydropyridine nicardipine on NREM sleep. We monitored *in vivo* calcium transients in cortical neurons and observed the duration and intensity of NREM sleep following intracerebroventricular (ICV) treatment in mice. Despite robust suppression of cortical calcium influx, dihydropyridine treatment did not significantly affect NREM sleep.

## Materials and methods

### Animals and surgeries

#### Animals

Experimental procedures were carried out in accordance with local and national regulations and after approval by the animal care and use committee of the University of Tsukuba. C57BL/6J male mice, 8–25 weeks old (Jackson Laboratory, Japan; *N* = 7), were used for sleep recordings. For *in vivo* calcium imaging, we crossed Ai148 (TITL2-GC6F-ICL-tTA2) mice (Allen Institute, WA, USA) with mice expressing Cre under the CaMKII promoter (Tsien et al., 1996), which led to the expression of GCaMP6f in forebrain excitatory neurons (*N* = 3). Mice were singly housed after surgery in a 12-h light and 12-h dark cycle with food and water *ad libitum*. Zeitgeber time 0 (ZT0) is light onset.

#### Electrode implantation

Mice were anesthetized with isoflurane (3–4% for induction and 2–2.5% for maintenance) and placed in a stereotaxic frame (David Kopf Instruments). The body temperature was maintained at 34–35°C with a heating pad. The scalp was removed, and the skull surface was cleaned with a scalpel blade. Two EEG screw electrodes were inserted into the skull for sleep recording. Two wire-electrodes (Cooner Wire, CA, USA) were implanted into the neck muscles bilaterally for electromyogram (EMG) recordings. Electrodes were connected to a standard 0.1-inch four-pin connector. This connector was fixed to the skull with cyanoacrylate and dental cement.

#### Microdrive implantation

Surgery followed with a previously published protocol (Matsumoto et al., 2020), briefly: tetrodes, EEG screws, and EMG wires were implanted using a custom-built microdrive. Mice were anesthetized with isoflurane; the surface of the brain was exposed by scalp incision followed by craniotomy. The dura was removed, and a microdrive system with six tetrodes (KANTHAL Precision Technology, nichrome, 14 μm in diameter) was implanted together with the EEG screws and EMG wires (Cooner Wire, CA, USA). Three tetrodes were placed in layer V of the primary motor cortex (M1) of the right and left hemispheres, and one tetrode into the ventral hippocampal commissure.

#### Intracerebroventricular cannula implantation

A single guide cannula of 27 gauge (RWD Life Science, CA, USA) was inserted with a tip distance of 1.9 mm from the surface of the brain at a 10° angle, targeting the lateral ventricle. During the ICV injection, injector cannulas of 33 gauge (RWD Life Science, CA, USA) were inserted through the guide cannula.

#### Optical window insertion

Optical windows were inserted into the skulls of three animals after EEG and EMG electrode implantation and ICV cannula insertion. A craniotomy was performed, covering the M1 and secondary somatosensory (S2) regions of the cortex using a dental drill. A hooked needle was used to remove a circular (2 mm) flap of the skull, leaving the dura intact. A Lipidure-CM5206 (NOF corporation) treated circular glass coverslip was held against the brain surface and glued to the skull with ultraviolet light hardening glue (BONDIC Starter Kit Complete; BONDIC, NY, USA). A custom-made metal head plate with a sufficient central opening was then secured to the skull with dental cement (SuperBond C&B set; Sun Medical, Shiga, Japan).

### Pharmacology

Mice were injected ICV with a 10% sorbitol vehicle solution (Sigma-Aldrich, USA) dissolved in ultrapure H_2_O (MilliporeSigma, Sigma-Aldrich, Inc., USA) or with 100 nM of nicardipine hydrochloride (N7510, Sigma-Aldrich, USA) dissolved in vehicle solution. During the ICV injection, an injector cannula of 33G was inserted through the guide cannula to within 0.1 mm of the tip of the guide cannula. The solution was carried *via* polyethylene tubing (RWD Life Science, CA, USA) from a 1 ml syringe (Terumo, Japan).

### Sleep recording and analysis

#### Recording of electrical signals

EEG and EMG electrodes were connected to a low-noise amplifier and digitizer board (Intan Technologies, CA, USA). Signals were recorded at a sampling rate of 1 kHz with the provided open-source software (Intan Technologies, USA). EEG and EMG were extracted, and band-pass filtered (EEG 0.1–500 Hz, EMG 10–500 Hz) using MATLAB (Matlab 2018a, The MathWorks, Inc., MA, USA) and provided import functions (Intan Technologies, CA, USA). After the electrode implantation, mice were given time to recover and acclimatize to recording chambers for 7 days, followed by 3 days of baseline recordings, 1 day of vehicle injection, followed by 1 day of nicardipine injection, with one rest day between injections.

For EEG and EMG recording during imaging, the signals were band-pass filtered and amplified using an analog amplifier (MEG-5200, NIHON KOHDEN, Japan), digitized at 2000 Hz (Digidata 1440A, Molecular Devices, USA), and acquired using Clampex 10.3 (Molecular Devices, USA). Each scan was digitized and recorded to match the imaging data.

The procedure for habituating the animals after tetrode implantation and obtaining neuronal spiking data, EEG, and EMG recordings followed published methods (Matsumoto et al., 2020).

#### Sleep deprivation

Mice were deprived of sleep by gentle handling and cage changes every hour for 4 h (Suzuki et al., 2013). Sleep deprivation started at ZT0 (onset of the light period). Mice were allowed to fall asleep undisturbed after sleep deprivation.

#### Sleep scoring

For sleep scoring, EEG and EMG signals were divided into regular-spaced epochs. Epoch length was set to 4 s for EEG data obtained during *in vivo* imaging because of the needed high resolution. Epochs of 10-s length were used for all other sleep analyses. Each epoch was scored as either wake, NREM, or REM sleep. EEG signals underwent fast Fourier transformation by custom MATLAB scripts (MATLAB 2018a, The MathWorks, Inc., MA, USA). Scoring was based on the power in the EEG delta band (0.5–4 Hz), the EEG theta band (6–10 Hz), and the ratio of delta to theta band power, as well as the integral of the EMG signal. Epochs with a high integral of EMG signal and low delta power were scored as wake. Epochs with a low integral of EMG signal and high delta power were scored as NREM. Epochs with a very low integral of EMG signal and a high theta to delta power ratio were scored as REM. The scoring was conducted using custom MATLAB programs (MATLAB 2018a, The MathWorks, Inc., MA, USA) (Bjorness et al., 2016).

#### Spike sorting

Tetrode signals were recorded with a Neuralynx Digital Lynx system using Neuralynx Cheetah software (Neuralynx, MT, USA) (Matsumoto et al., 2020). Spikes were detected online, and the multi-unit activity was sorted into single-unit activity offline with Spike Sort 3D software (Neuralynx, MT, USA). Cluster classification was performed using previous methods (Matsumoto et al., 2020). Briefly, clusters were considered to represent single units if the isolation distance was ≥ 20 and L-ratio ≤ 0.3. Only units with a peak-to-peak width greater than 250 μs were classified as putative excitatory neurons and were used for this study.

### *In vivo* two-photon imaging and analysis

After the optical window surgery, mice were given 7 days to recover/habituate to the head plate. For imaging, they were placed on an air-suspended spherical treadmill. The mice could undergo spontaneous wake and NREM transitions on the spherical treadmill during imaging. Due to the short recording duration of 2 h or less, we did not reliably observe REM episodes. The calcium activity was observed with a two-photon microscope (Axio Examiner Z1, Zeiss) (Miyazaki et al., 2020). Imaging of GCaMP6f expressing neurons was conducted in cortical layer II/III, with excitation at 910 nm with a Ti: Sa laser (Mai Tai DeepSee, Spectra-Physics). For each session, 1,400 frames were acquired at a rate of 440 ms per scan, which took about 11 min. In total, there were 13 sessions per animal: three sessions for baseline, three sessions after vehicle injection, and seven sessions after nicardipine injection.

The scanned images were loaded into a custom-made MATLAB program (MATLAB 2018a, The MathWorks, Inc., MA, USA), where they were analyzed semi-automatically. The extracted raw data were motion corrected (Pnevmatikakis and Giovannucci, 2017). Regions of interest (ROI) were manually drawn to match the somata of the neurons. Their calcium activity was detected as a change in fluorescence over baseline fluorescence traces (dF/F), following this function: dF/F = (F_*t*_–F_0_)/F_0_, where F_0_ is the baseline mean of the four frames previous to the F_*t*_ frame. The integral of dF/F over the 0.5 thresholds was calculated to compare the calcium activity among different conditions. To determine the threshold, we calculated the average standard deviation of the dF/F signal under nicardipine for all neurons when the relative contribution of calcium transients to noise was the lowest. The value of 0.5 represents two standard deviations above the mean (rounded from a value of 0.502). Thus, the thresholding removes the background noise and shows only the difference in calcium activity during baseline, vehicle, and nicardipine sessions. The temporal resolution of our system (440 ms per scan) and the decay time constant of the indicator are insufficient (Chen et al., 2013; Umpierre et al., 2020; Huang et al., 2021; Zhang et al., 2021) for a proper analysis of underlying spiking patterns. The average of the above 0.5 dF/F integrals of the three baseline scans was set to 100%. The integral obtained during scans following vehicle injection and nicardipine injection was normalized to that value. Scans were obtained 40 min after vehicle injection and 100 min after nicardipine injection.

The statistical analyses were performed in MATLAB (MATLAB 2018a, The MathWorks, Inc., MA, USA) and IBM SPSS Statistics (SPSS for Windows, Version 25.0. Armonk, NY: IBM Corp.) for two-way repeated measures ANOVA. Statistical significance was defined as *p* < 0.05. Results are presented as mean ± standard error of the mean (SEM) unless otherwise noted.

## Results

In this study, we could assess *in vivo* calcium dynamics together with EEG/EMG recordings in mice freely transitioning between vigilance states. These measures provide optimal quantitative measures for the potential role of L-type VGCC-mediated neuronal calcium influx in sleep function.

### Calcium influx into excitatory neurons is larger in non-rapid eye movement sleep compared to waking

**FIGURE 1:**
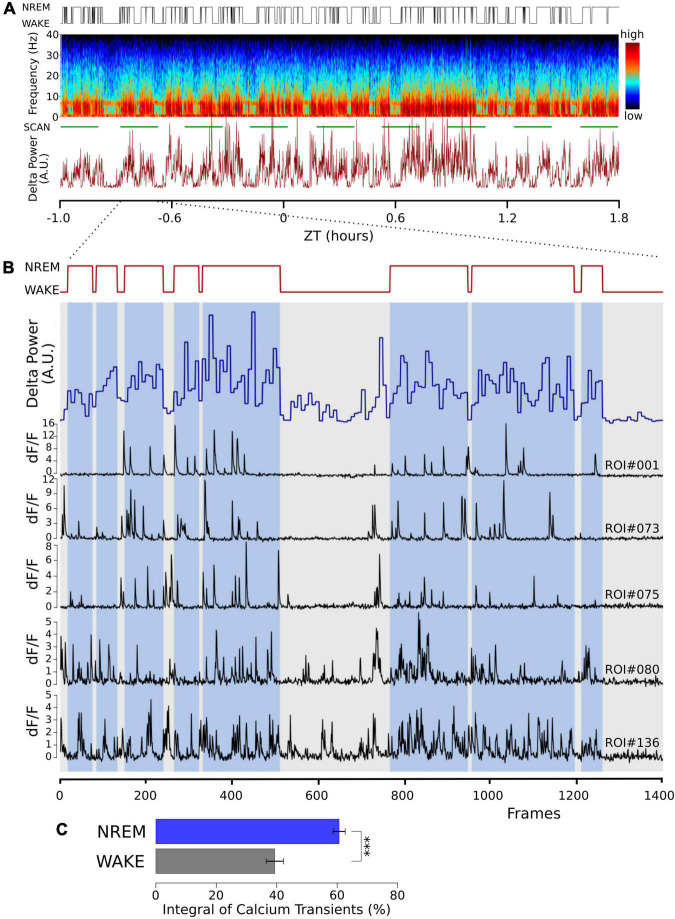
Cortical calcium transients during waking and NREM sleep. Somatic calcium transients are larger in NREM sleep compared to waking in simultaneous EEG and EMG recordings and *in vivo* two-photon GCaMP6f calcium imaging. **(A)** Sample of one recording session. Top trace: hypnogram derived from the simultaneously recorded EEG and EMG. Middle graph: Pseudo-colored spectrogram of the EEG recording. The green line below indicates the time during which the imaging data was obtained (SCAN). The graph below shows power in the EEG delta band (0.5–4 Hz) over time. **(B)** Enlarged time window from panel **(A)** (indicated by the dotted lines), representing one imaging episode of 1,400 frames lasting 11 min. Top trace: Hypnogram, times in NREM sleep are marked with blue bars, showing two long wake episodes. Blue trace: EEG delta power in 4 s epochs. Black traces: sample of dF/F traces from five different regions of interest (ROI), drawn over somata of layer II/III excitatory neurons. Notice the decrease in activity during the wake episodes. **(C)** Average data from a total of 100 neurons in 3 mice are shown (mean ± SEM). The dF/F traces were thresholded at 0.5 for all ROIs (see section “Materials and methods”), and the integral over the threshold was calculated. Given the calcium fluorescence variability between ROIs, we calculated the activity in its percentage during NREM and its percentage during wakefulness, corrected by the relative durations of NREM and wakefulness. *P* values less than 0.001 are indicated with three asterisks.

Burst firing is an effective way to produce large intracellular calcium transients. In shorter recordings (Ohyama et al., 2020), we have shown that the propensity for burst firing is larger in NREM sleep compared to waking. In 24 h recordings in which the animals naturally transitioned between vigilance states, we again found that burst frequency in NREM was significantly larger than in waking (NREM = 0.362 ± 0.14 min^–1^; waking = 0.295 ± 0.12 min^–1^, ten neurons, *t* = -3.502, *p* < 0.007, paired, two-tailed *T*-test). This is compatible with larger calcium transients in NREM sleep; we observed intracellular calcium transients directly to further explore this. Using *in vivo* two-photon imaging of the genetically encoded calcium sensor GCaMP6f, we observed calcium signals in naturally occurring wake and NREM sleep in head-fixed animals that could freely walk on air-suspended Styrofoam balls. Calcium influx into excitatory neurons was larger during NREM sleep compared to waking (Figure 1).

The higher calcium transients in NREM sleep are compatible with previously published results (Tatsuki et al., 2016, 2017; Shi and Ueda, 2018), indicating that intracellular calcium transients contribute to the characteristic ON/OFF oscillation in NREM sleep. We, therefore, wanted to test the effects of acutely perturbing calcium influx into cortical neurons and measure the effect on NREM sleep.

### Intracerebroventricular nicardipine reduces calcium influx into excitatory neurons

**FIGURE 2:**
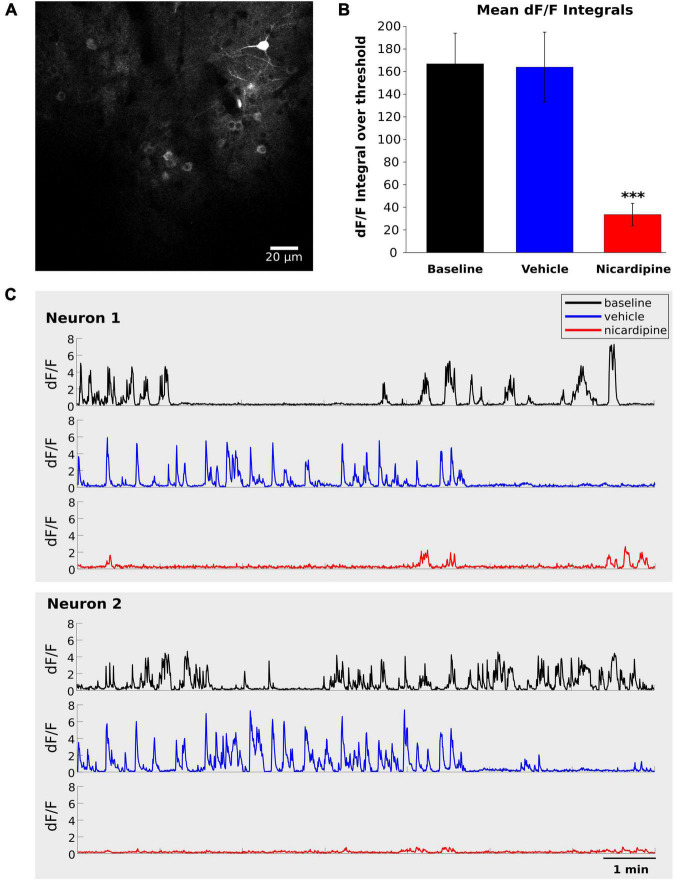
Intracerebroventricular application of Nicardipine reduces cortical somatic calcium transients. Layer II/III neuron somatic calcium transients in *in vivo* two-photon imaging of GCaMP6f under baseline conditions, after ICV vehicle injection, and after ICV nicardipine injection. **(A)** Representative grayscale image of a field of view from one experiment. **(B)** Comparison of calcium signals under baseline conditions (black), after 40 min of ICV vehicle injection (blue), and after 100 min of ICV nicardipine injection. A threshold of 0.5 was applied to the dF/F signals (see section “Materials and methods”), which were then separately integrated over time for the three conditions. (*N* = 3 animals; 63 neurons, 24 neurons, and 13 neurons), error bars are SEM. *P* values less than 0.001 are indicated with three asterisks. **(C)** Representative dF/F traces over time of two neurons in the three conditions (black, baseline; blue after vehicle injection; red after nicardipine injection).

Therefore, we next investigated the inhibition of calcium activity under the influence of nicardipine, a dihydropyridine L-type VGCC blocker, using *in vivo* two-photon calcium imaging of GCaMP6f labeled neurons. The calcium activity of individual neurons decreased significantly 100 min after 100 nM ICV nicardipine injection (Figure 2). We calculated the integral over time of the dF/F signals above a 0.5 dF/F threshold across three animals. As shown in Figure 2B, this integral was significantly reduced under nicardipine, indicating that it successfully blocked calcium entry through L-type VGCCs (Wu et al., 1999). As expected (Wu et al., 1999), nicardipine did not eliminate calcium transients (Figure 2C), indicating that not all calcium influx was blocked.

We have thus shown that activity-dependent calcium influx into cortical neurons is significantly reduced by ICV nicardipine application. We next investigated the effects of such reduced calcium influx on NREM sleep, including the generation of EEG delta waves, and on the efficiency with which the need for sleep was resolved, as indicated by the reduction of EEG delta power during NREM sleep.

### Intracerebroventricular nicardipine has no significant effect on non-rapid eye movement sleep

To observe an impairment in NREM sleep, we administered vehicle or nicardipine *via* ICV injection under three different sleep conditions and at different zeitgeber times (ZT). First, at ZT0, when mice are at their normal peak sleep need. Second, at ZT12, when sleep need is typically lowest, and third, after four h of sleep deprivation starting at ZT0, resulting in very high sleep need at ZT4.

**FIGURE 3:**
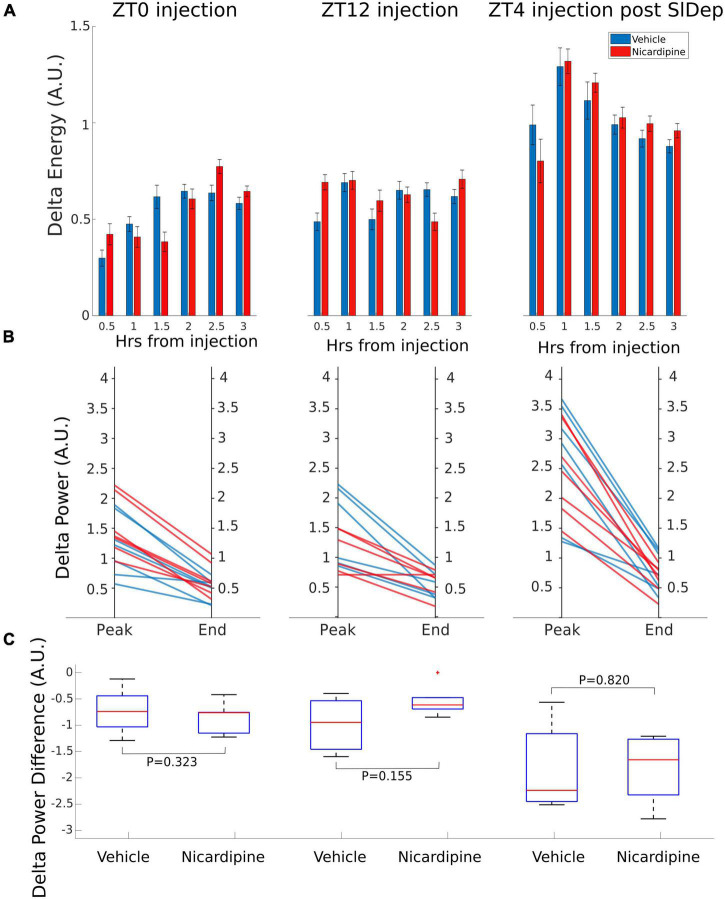
Application of Nicardipine ICV does not Affect EEG Delta Oscillations. The power of EEG oscillations in the delta (0.5–4 Hz) band during NREM sleep did not differ when the animals received ICV vehicle injections or ICV nicardipine injections. **(A)** Power in the EEG delta band during NREM sleep is integrated over time to obtain delta energy. Delta energy production was measured for 3 h in three different conditions [at ZT0, at ZT12, and at ZT4 after 4 h of sleep deprivation (SlDep)]. Each animal was measured after vehicle injection (blue) and after nicardipine injection (red). Delta energy is plotted over time in 0.5-h bins. Nicardipine did not affect EEG delta energy in any of the three conditions at any time point. **(B)** The effectiveness of NREM sleep episodes can be measured by the reduction in EEG delta power over the duration of the episode. We analyzed NREM sleep episodes longer than 5 min in the recordings from ZT0, ZT12, and ZT4 after sleep deprivation. Peak EEG delta power within the first 3 min is plotted on the left-hand side, and the last epoch’s delta power is plotted on the right-hand side. After both vehicle treatment (blue) and nicardipine treatment (red), EEG delta power was lower at the end of the episode compared to the beginning. **(C)** Box plots (orange line, mean; blue box 25th percentile; black whiskers 90th percentile) of comparison between vehicle treatment and nicardipine treatment on the average reduction in delta power during NREM sleep episodes longer than 5 min. In all three conditions, there was no significant difference between vehicle and nicardipine. *P*-values were obtained with paired two-tailed Student’s *t*-test (*N* = 7).

There was no difference in NREM EEG delta (0.5–4 Hz) energy between the two treatment conditions, nicardipine, compared to control injections, both immediately after the injection and a few hours later, when the nicardipine effect had dissipated (Figure 3A).

Therefore, there was no correlation between the generation of NREM sleep EEG delta oscillations (Supplementary material Appendix 1 and Supplementary Figure 1) and the drastic reduction of activity-dependent calcium influx in cortical neurons. Moreover, sleep-need reduction during NREM sleep episodes (Bjorness et al., 2016) was not affected by the reduced calcium influx (Figures 3B,C and Table 1). The reduction in the power of EEG delta oscillations during NREM episodes was not different between treatment conditions (Figure 3C).

**TABLE 1:** Means of NREM delta power decay peaks and ends during NREM sleep episodes.

	Peak			End		
	Vehicle	Nicardipine	*P*-value	Vehicle	Nicardipine	*P*-value
ZT0	1.21 (0.195)	1.516 (0.180)	0.986	0.486 (0.07)	0.654 (0.106)	0.686
ZT12	1.50 (0.269)	1.127 (0.139)	0.182	0.531 (0.99)	0.573 (0.092)	0.746
ZT4	2.62 (0.360)	2.510 (0.087)	0.38	0.825 (0.135)	0.674 (0.087)	0.115

NREM sleep episodes longer than 5 min were selected in three different sleep conditions (ZT0, ZT12, and ZT4 after sleep deprivation). The peak delta power value during the initial 3 min of each sleep episode and the end delta power value was calculated and averaged. Means (SEM) of a total of 7 animals are indicated. The *p*-values were obtained with paired two-tailed Student’s *t*-test.

We also investigated the sleep patterns of the mice under different treatment conditions by measuring the time spent in wake, NREM sleep, and REM sleep. We did not observe any significant difference between nicardipine and vehicle-treated conditions in the overall durations of wake, NREM sleep, and REM sleep (Table 2 and Supplementary Figure 2).

**TABLE 2:** Mean number of NREM episodes and mean NREM episode durations.

	Number of NREM episodes			NREM episode duration (min)		
	Vehicle	Nicardipine	*P*-value	Vehicle	Nicardipine	*P*-value
ZT0	116.143 (5.535)	109.857 (10.243)	0.552	19.357 (0.923)	18.301 (1.71)	0.3
ZT12	65.667 (3.221)	80 (14.507)	0.402	10.944 (0.537)	13.333 (2.418)	0.296
ZT4	100.714 (11.924)	77 (8.083)	0.051	16.786 (1.987)	12.83 (1.347)	0.07

The same NREM sleep episodes from Table 1 were selected in three different sleep conditions (ZT0, ZT12, and ZT4 after sleep deprivation). The number of NREM episodes and their durations were compared (*N* = 7). Mean values and SEM values in parentheses are indicated. The *p*-values were obtained with paired two-tailed Student’s *t*-test. This shows that the episode length and durations were not significantly different between vehicle and nicardipine conditions.

In summary, ICV injection of nicardipine drastically reduced activity-dependent calcium influx into cortical neurons but did not affect the generation of NREM EEG delta oscillations or the dissipation of sleep need during NREM sleep.

## Discussion

In this study, we demonstrated a strong reduction of calcium influx into cortical neurons after applying the L-type VGCC blocker nicardipine *in vivo*. This reduced calcium influx did not interfere with the generation of EEG delta waves and did not block the gradual reduction of EEG delta power during NREM sleep. NREM EEG delta power has been studied as a strong indicator of sleep need (Borbély and Achermann, 1999; Dijk, 2009). However, the link between sleep need and the cortical ON/OFF oscillation that underlies the generation of delta oscillations and cortical activity is not well elucidated. One compelling argument posits that calcium activity is involved in generating delta oscillations (Tatsuki et al., 2016, 2017; Shi and Ueda, 2018). Thus, we sought to investigate the role of activity-dependent calcium influx through L-type VGCCs using pharmacological block by nicardipine. L-type VGCCs are widely expressed in cortical neurons, and 50% of action potential-driven postsynaptic calcium influx is mediated by L-type VGCCs (Wu et al., 1999). A dihydropyridine blocker was selected since they are a widely used class of drugs that readily penetrate the blood-brain barrier and might have side effects that have been overlooked so far.

In agreement with previous results, we found higher incidences of burst firing (Hubel, 1959; Hobson and McCarley, 1971; Ohyama et al., 2020) and larger calcium transients (Ohyama et al., 2020) in cortical neurons in NREM sleep compared to waking. Tetrode recordings lasting 24 h show that increased bursting is a stable characteristic of NREM sleep. Some groups have reported lower calcium activity in cortical neurons in NREM sleep compared to waking (Niethard et al., 2016, 2018). However, a complex picture emerges with different cell types and compartments showing different changes in calcium activity between waking and NREM sleep (Sigl-Glöckner and Seibt, 2019). At this stage, we cannot fully resolve the differences between our findings and some prior results, but previous studies have found increased calcium activity in subtypes of excitatory cortical neurons (Niethard et al., 2016, 2021).

It was recently proposed that increased burst firing and the related calcium influx were fundamental for generating ON/OFF state oscillations and NREM sleep (Tatsuki et al., 2016, 2017; Shi and Ueda, 2018). Our results do not support an absolute need for activity-driven calcium influx in the generation of EEG delta waves because, as aforementioned, a large percentage of the calcium influx is through L-type VGCCs. Alternative sources of intracellular calcium might still play a role. Other VGCCs such as N, P/Q or T-type, NMDA receptors, or phospholipase C signaling are other possible sources for activity-dependent calcium entry. Calcium influx through T-type channels was shown to characterize high-frequency bursts of action potentials during NREM sleep and to initiate UP states (Crunelli et al., 2014). Because of their presynaptic physiology—coupling neuronal excitation to the secretion of neurotransmitters (Fletcher et al., 2001; Wormuth et al., 2016), investigation of other VGCCs remains challenging. Several studies have shown that systematic application of the NMDA receptor blocker ketamine results in particularly intense EEG delta oscillations (Fontanini et al., 2003; Chauvette et al., 2011; Ahnaou et al., 2017), arguing against disruption of ON/OFF oscillations and delta waves by blocking neuronal calcium influx. Thus, an obligatory link between EEG delta oscillations and calcium influx is unlikely. Further investigations into the calcium activities of different receptors and signaling processes during the ON/OFF phases are necessary.

A more fundamental and less addressed issue concerns the potential down streams signals generated by delta wave activity mediating sleep need resolution. Does neural activity underlying delta waves contribute to sleep need reduction? If so, how? In our study, L-type VGCC blockade produced a substantial reduction of calcium transients; nevertheless, the decay of delta wave activity was unchanged from the control. Thus, calcium influx does not link neuronal activity and sleep need resolution. However, we would like to emphasize the need to investigate other potential links between EEG delta power and sleep need resolution.

This might be achieved by the recent advances in the proteomic and transcriptomic investigation of sleep, which provide us with good markers for sleep need buildup and resolution (Bjorness et al., 2009, 2016; Wang et al., 2018; Suzuki et al., 2020). For example, wake-expressed c-fos levels rapidly drop during subsequent NREM sleep (Basheer et al., 1999), and reduced levels of immediate early genes may constitute a signaling pathway linking NREM delta power to changes in gene expression patterns. In the future, these markers can be used to determine whether NREM delta power is causally related to sleep need resolution or is an epiphenomenon of the process.

Our results show that neuronal firing and calcium activity increase during NREM sleep compared to wakefulness. Acute inhibition of calcium influx through L-type VGCC did not affect EEG delta power or sleep need resolution. This result agrees with the clinical literature on dihydropyridines, which readily cross the blood-brain barrier. Clinical studies showed no patients complaining of side effects in terms of mood and sleep (Leonetti and Salvetti, 1994; He et al., 2018). A recent clinical study showed that hypertensive patients receiving the dihydropyridine amlodipine less frequently suffered from insomnia than when they received other medications (Isaveya et al., 2020). In light of the proposed importance of calcium transients in NREM sleep and NREM sleep delta power (Tatsuki et al., 2016, 2017; Shi and Ueda, 2018), the lack of significant effects on NREM sleep of even high doses of dihydropyridines is reassuring for the large number of patients who take these substances as a daily medication.

## Data availability statement

The original contributions presented in this study are included in the article/Supplementary material, further inquiries can be directed to the corresponding author.

## Ethics statement

The animal study was reviewed and approved by the animal care and use committee of the University of Tsukuba.

## Author contributions

RG and KV contributed to the conception and design of the study. JD performed the statistical analysis. SM contributed to the database. GH performed the experiments and wrote the first draft of the manuscript. All authors contributed to manuscript revision, read, and approved the submitted version.

## Supplementary material

Supplementary Figure 1 Sample EEG Delta Power and Hypnogram Plots over 24 h for Vehicle and Nicardipine Treatment. Power in the EEG delta (0.5–4 Hz) band (top) and hypnogram (bottom) from one mouse plotted against time for two non-consecutive 24 h recordings. Light (inactive) phase from ZT0 to ZT12 and dark (active) phase from ZT12 to ZT24. The graph on the left is the vehicle condition (indicated by the blue vertical bar), and the graph on the right is the nicardipine condition (indicated by the yellow vertical bar). **(A)** Injection of vehicle and nicardipine at ZT0. **(B)** Injection of vehicle and nicardipine at ZT 12. **(C)** Injection of vehicle and nicardipine at ZT4 after 4 h of sleep deprivation from ZT0 to ZT4.

Supplementary Figure 2 Vigilance State Plots at 1-h Intervals for Vehicle and Nicardipine Treatment. Plots of vigilance state duration in minutes per 1 h bin (top, wake; middle, NREM sleep; bottom, REM sleep) over 24 h. Data is mean ± SEM for *N* = 7 animals. ZT0–ZT12 is the light (inactive) phase, and ZT12–ZT24 is the dark (active) phase. Data from ICV vehicle treatment in orange, from ICV nicardipine treatment in blue. Treatment is indicated by the gray vertical bar. **(A)** Treatment time ZT0. **(B)** Treatment time ZT12. **(C)** Treatment time ZT4 after sleep deprivation from ZT0 to ZT4. *P*-values on top of graphs are from two-way repeated measures ANOVA for treatment effect. There was no significant difference between treatment conditions for any vigilance state.
