# Supplementary figures and images for: Dihydropyridine calcium blockers do not interfere with non-rapid eye movement sleep
Source: Front Neurosci. 2022 Oct 19;16:969712. doi: 10.3389/fnins.2022.969712 (PMC9626806; doi:10.3389/fnins.2022.969712)

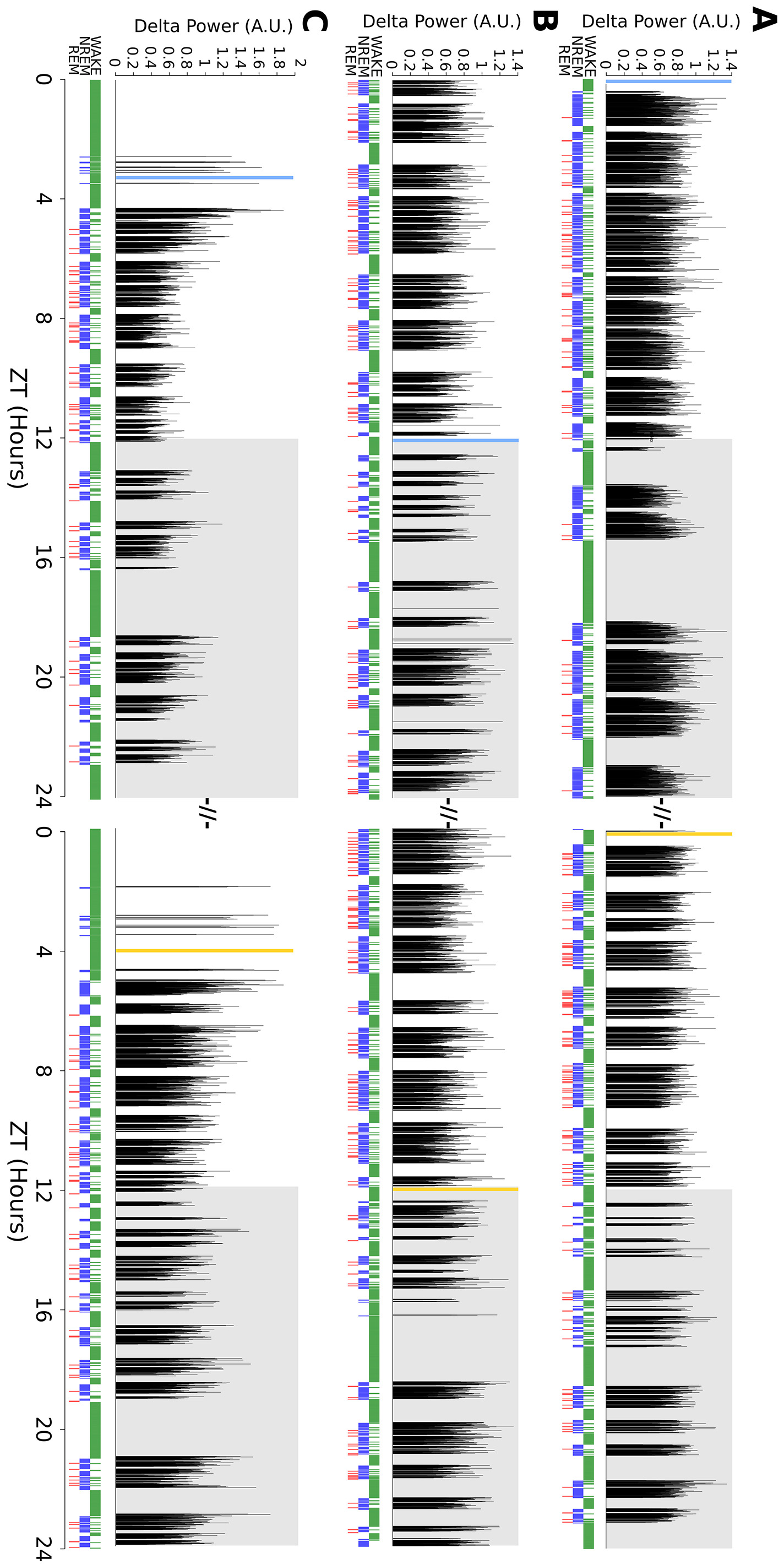

Supplement: Supplementary Figure 1 — Sample EEG Delta Power and Hypnogram Plots over 24 h for Vehicle and Nicardipine Treatment. Power in the EEG delta (0.5–4 Hz) band (top) and hypnogram (bottom) from one mouse plotted against time for two non-consecutive 24 h recordings. Light (inactive) phase from ZT0 to ZT12 and dark (active) phase from ZT12 to ZT24. The graph on the left is the vehicle condition (indicated by the blue vertical bar), and the graph on the right is the nicardipine condition (indicated by the yellow vertical bar). (A) Injection of vehicle and nicardipine at ZT0. (B) Injection of vehicle and nicardipine at ZT 12. (C) Injection of vehicle and nicardipine at ZT4 after 4 h of sleep deprivation from ZT0 to ZT4. [file Image_1.JPEG]

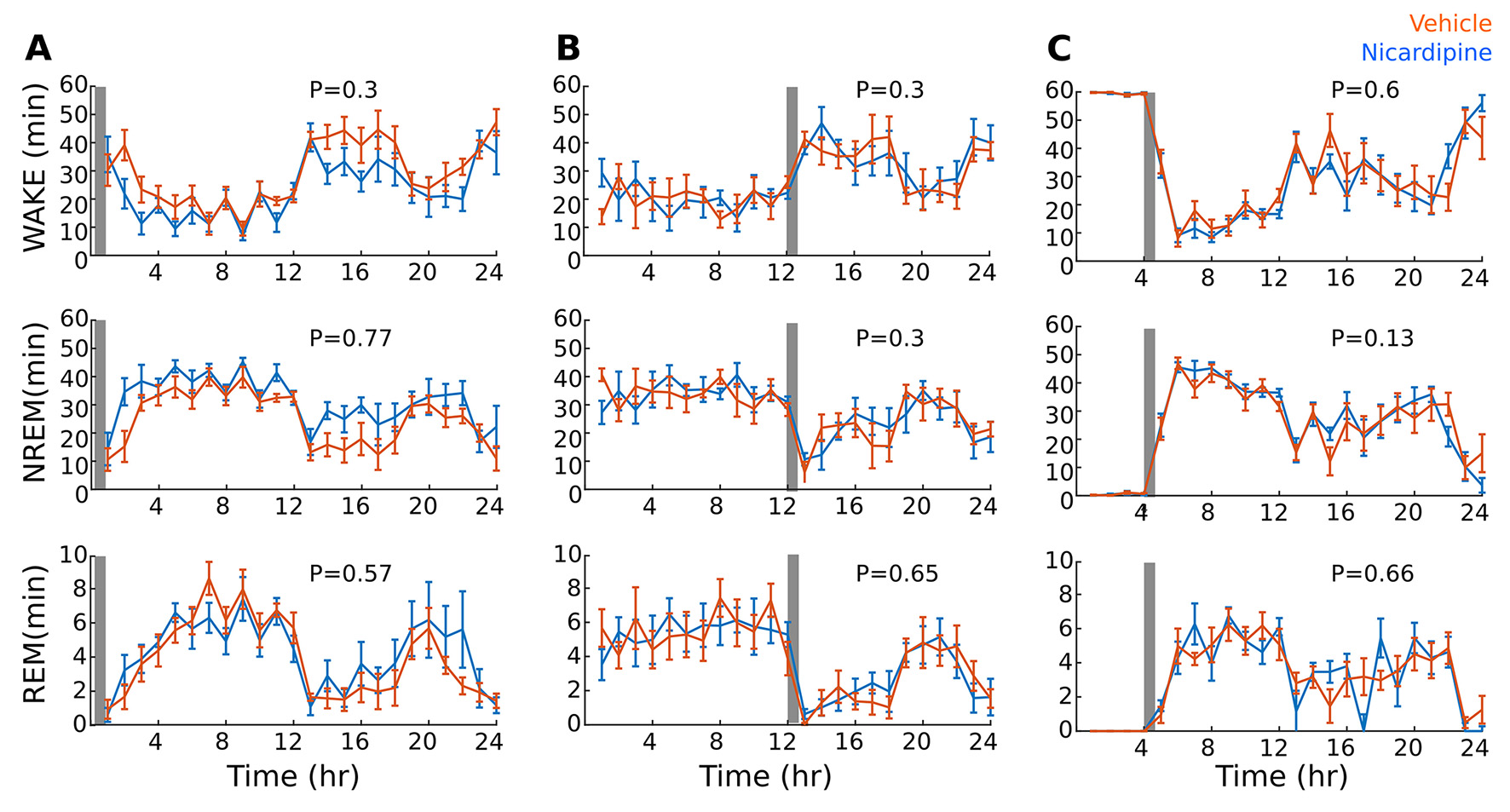

Supplement: Supplementary Figure 2 — Vigilance State Plots at 1-h Intervals for Vehicle and Nicardipine Treatment. Plots of vigilance state duration in minutes per 1 h bin (top, wake; middle, NREM sleep; bottom, REM sleep) over 24 h. Data is mean ± SEM for N = 7 animals. ZT0–ZT12 is the light (inactive) phase, and ZT12–ZT24 is the dark (active) phase. Data from ICV vehicle treatment in orange, from ICV nicardipine treatment in blue. Treatment is indicated by the gray vertical bar. (A) Treatment time ZT0. (B) Treatment time ZT12. (C) Treatment time ZT4 after sleep deprivation from ZT0 to ZT4. P-values on top of graphs are from two-way repeated measures ANOVA for treatment effect. There was no significant difference between treatment conditions for any vigilance state. [file Image_2.JPEG]
